# Tin-Foil as a Filling-Material

**Published:** 1894-06

**Authors:** Benjamin Lord

**Affiliations:** New York


### TIN-FOIL AS A FILLING-MATERIAL.[1]

BY DR. BENJAMIN LORD, NEW YORK.

Mr. President and Fellow-Members,—The subject of the use of tin-foil as a filling-material is one, to my mind, of peculiar interest and importance; and the attention that it has received during the last six months is most encouraging, and promises much, I believe, in the treatment of the teeth in the future.

A paper was read a week ago before the First District Society having this title: "Has not Tin-Foil as a Material for filling Teeth been overrated?"

I was very sorry not to be able to attend that meeting, to hear the paper read and listen to the discussions upon it. I do not know what views were advanced or what arguments were used to show that the value of tin-foil had been overrated; but, from my experience and observation, I do not understand that its merits or value can be overrated.

Now, those who place this high estimate upon tin-foil should have some good reasons for it.

It is said that we know the world, or learn the world, by comparison. If we compare tin-foil with gold-foil, we find that the tin, being softer, works more kindly, and can be more readily and with more certainty adapted to the walls, the inequalities, and the corners of the cavities.

We find also that tin welds—mechanically, of course—more surely than soft gold, owing to its greater softness; the folds can be interlaced or forced into each other, and united with more certainty, and with so much security that, after the packing and condensing are finished, the mass may be cut like molten metal.

I contend, moreover, that for contouring the filling or restoring the natural shape of the teeth, where there are three walls remaining to the cavity, tin is fully equal to gold, and in some respects even superior; as tin can be secured, where there is very little to hold or retain the filling, better than gold, owing to the ease and greater certainty of its adaptation to the retaining points or edges of the cavity.

It will be said, however, that tin fillings will wear away. The surfaces that are exposed to mastication undoubtedly will wear in time; but the filling does not become leaky, if it has been properly

---

[1] Read before the New York Odontological Society, March 20, 1894.

packed and condensed, nor will the margins of the cavity be attacked by further decay on that account.

Altogether I believe that we can make more perfect fillings with tin than we can with gold, taking all classes of cavities; but it must not be understood that it is proposed that tin should ever take the place of gold where the circumstances and conditions indicate that the latter should be used. Of course, the virtue is not in the gold or in the tin, but in the mechanical perfection of the operation, and tin having more plasticity than gold, that perfection can be secured with more ease and certainty.

If we compare tin with amalgam, we must certainly decide in favor of the former and give it preference; as if it is packed and condensed as perfectly as it may be, we know just what such fillings will do every time. We know that there will be no changes or leakage of the fillings at the margins; whereas, with amalgam, the rule is shrinkage of the mass, and consequently the admission of moisture around the filling, the result being further decay. It is not contended that this is always the result with amalgam, but it is the general rule; yet we must use amalgam, as there are not a few cases where it is the best that we can do; but it is to be hoped, and I think it may be said, that as manipulative skill advances, amalgam will be less and less used. For so-called temporary work, very often I prefer tin to gutta-percha, as it makes a much more reliable edge and lasts longer, even when placed and packed without great care.

Gutta-percha is not infrequently left too long, and the surface of the filling becomes worn or softened, thus leaving the margins of the cavities exposed to further decay; and often, when we come to fill permanently, we find that the cavities are much larger than when we saw them first. I have felt that there was quite a good deal of risk to the best interests of our patients in leaving gutta-percha in the teeth very long, owing to this fact and to the uncertainty and almost impossibility of making perfect margins; yet it is an invaluable material in the treatment of the teeth.

I may say that I think we are now having a better article of gutta-percha than we have had hitherto,—that which is called dentron; it has been brought out by Mr. T. B. Owens, of Boston. The most important quality that I have yet noticed in it is that a much more perfect edge can be made with it, owing to its greater density.

To get the best results from the use of tin as a filling-material two or three considerations are imperative. First, we must have good foil. I have almost feared that the art of making the best tin-foil was, at least in some measure, lost. I have brought to the meeting some of a lot that Mr. R. S. Williams made about twenty-five years ago, to show to any who may be interested to see it. I have made use of the same lot ever since, and do not observe any change or deterioration in it. I have tried the tin made by Mr. Williams and various other makers since, and have never met with any so tough and strong as this.

Mr. E. Kearsing, of Brooklyn, says that he has developed a new method of making tin-foil. I have tried it, and found it very soft and very nice; the best, I may say, that I have ever used, except that of which I have spoken, which I have been using so long.

Then, secondly, it is very necessary to have suitable instruments. I do not think that the best work can be made with the instruments that are in general use, even by trained hands. If any one does not believe that tin-foil can be made to do all that I have said of it, I would suggest or advise that he try what can be done; and the very best way to give it a trial is to have the teeth in one's own hand, and not in the mouth of a patient. Put the teeth into plaster; take the worst kind of cavities; have suitable instruments and a good foil, and you will soon find that you can do almost anything that you would like to in the way of filling a tooth.

I do not think that serrated points are as good or as suitable as a single point, or what might be called a diamond point, made so by a short bevel of the sides of the instrument at the tip. Be sure always to fold the foil into strips of suitable width for the cavity, and not to use it in rolls or pellets, or in any other form than strips.

Here I may relate an incident. Some twenty-five years ago, I said at a large meeting of dentists that I believed that if tin-foil were used in all cases, considering the hands that fill teeth, more teeth would be preserved; and the sentiment was responded to by the clapping of hands. The idea or thought was that tin-foil required less training and manipulative skill to use it successfully than gold.

The question very naturally arises, Why has not tin been used to a larger extent than it is? Probably one important reason is, because it is not gold, and the popular feeling is in favor of gold, not considering whether it is better or as good as tin.

Another reason may be that many dentists have not learned to use tin, and so use amalgam instead. If I understand correctly, tin is used very little, nor is its use much taught in the dental schools, which, if true, is a grievous mistake.

I have considered, I wish to say, that the "new departure" theory, which was started some sixteen years ago and advocated with a good deal of enthusiasm by some, did not encourage manipulative skill in the use of foils, as it brought plastics into use to such an unwarranted extent that the training of the mind and hands in the use of the former was likely to be neglected.

